# Inference, Prediction, & Entropy-Rate Estimation of Continuous-Time, Discrete-Event Processes

**DOI:** 10.3390/e24111675

**Published:** 2022-11-17

**Authors:** Sarah E. Marzen, James P. Crutchfield

**Affiliations:** 1 W. M. Keck Science Department of Pitzer, Scripps, and Claremont McKenna College, Claremont, CA 91711, USA; 2 Complexity Sciences Center and Physics and Astronomy Department, University of California at Davis, One Shields Avenue, Davis, CA 95616, USA

**Keywords:** Poisson process, renewal process, hidden semi-Markov process, hidden Markov chain, *ϵ*-machine, Shannon entropy rate, optimal predictor, minimal predictor, 02.50.-r, 05.45.Tp, 02.50.Ey, 02.50.Ga

## Abstract

Inferring models, predicting the future, and estimating the entropy rate of discrete-time, discrete-event processes is well-worn ground. However, a much broader class of discrete-event processes operates in continuous-time. Here, we provide new methods for inferring, predicting, and estimating them. The methods rely on an extension of Bayesian structural inference that takes advantage of neural network’s universal approximation power. Based on experiments with complex synthetic data, the methods are competitive with the state-of-the-art for prediction and entropy-rate estimation.

## 1. Introduction

Much scientific data is dynamic: rather than a static image, we observe a system’s temporal evolution. The additional richness of dynamic data offers improved understanding, but we may not know how to leverage the richer temporal data to yield new insights into a system’s behavior and structure.

For example, while there are extensive records of earthquake occurrence and magnitude, geophysics still cannot predict earthquakes well or estimate their intrinsic randomness [1]. Similarly, modern neurophysiology can identify which neurons spike when, but neuroscience still lacks a specification of the “neural code” that carries actionable information [2]. Furthermore, finally, we can observe many organisms in detail as they conduct their lives, but still are challenged to model their behavior [3,4].

These natural processes operate not only in continuous-time, but over discrete events—earthquake or not; neural spike or not; eating, sleeping, or roaming. Their observations belong to a finite set and are not better-described as a collection of real numbers. These disparate scientific problems and many others beg for methods to infer expressive continuous-time, discrete-event models, to predict behavior, and to estimate key system properties.

The following develops a unified framework that leverages the inferential and predictive advantages of the *unifilarity* of stochastic process models. This property means that a model’s underlying states—the *causal states* [5] or *predictive-states* [6]—can be uniquely identified from past data. We adapt the universal approximation power of neural networks [7] to this setting to model continuous-time, discrete-event processes. Said simply, the proposed model-inference algorithm is the continuous-time extension of *Bayesian structural inference* [8]. Altogether, this advances the state of the field of computational mechanics [5] by developing new discrete-event, continuous-time predictive model inference methods.

Using the *Bayesian information criterion* to balance model size against estimation error [9], we infer the most likely unifilar hidden semi-Markov model (uhsMm) [10] given data. This model class is more powerful than (“nonhidden”) semi-Markov models (sMms) in the sense that uhsMms can finitely represent continuous-time, discrete-event stochastic processes that cannot be represented as finite sMms. Moreover, with sMms emitted event symbols depend only on the prior symbol and their dwell times are drawn from an exponential distribution. With uhsMms, in contrast, the probability of emitted symbols depends on arbitrarily long pasts of prior symbols *and* event dwell times depend on general (nonexponential) distributions.

Beyond model inference, we apply the closed-form expressions of Ref. [10] to the inferred uhsMm to estimate a process’ entropy rate, removing statistical sampling approximations in this last step and markedly improving accuracy. Moreover, we use the inferred uhsMm’s causal states to predict future events in a given time series via a *k*-nearest neighbors algorithm. We compare the inference and prediction algorithms to reasonable continuous-time, discrete-event adaptations of current state-of-the-art algorithms. The new algorithms are competitive with state-of-the-art entropy rate estimation algorithms as long as model inference is in-class, meaning that the true model producing the data is equivalent to one of the models in our search.

Next, we review related work. Section 3 then introduces unifilar hidden semi-Markov models, while Section 4 shows that they are minimal sufficient statistics for prediction. Section 5 describes our new algorithms for model inference, entropy rate estimation, and time series prediction. We then test them on complex synthetic data—data from processes that are memoryful and exhibit long-range statistical dependencies. Finally, Section 6 discusses extensions and future applications.

## 2. Related Work

Many methods exist for analyzing discrete-time processes. The *autoregressive AR-k procedure*, a classical technique, predicts a symbol as a linear combination of previous symbols. A slight modification leads to the *generalized linear model* (GLM), in which the symbol probability is proportional to some function of a linear combination of previous symbols [11]. Previous approaches also use the *Baum-Welch algorithm* [12], *Bayesian structural inference* [8], or a nonparametric Bayesian approach [13] to infer a hidden Markov model or probability distribution over hidden Markov models of an observed process. Bayesian structural inference, which is most closely related to the method proposed here, enumerates all model topologies and calculates the probability of each model under the data, utilizing the unifilarity property (to be discussed) and conjugate priors to the greatest extent. If the most likely state of the hidden Markov model is correctly inferred, one can use the model’s structure (state and transition probabilities) to predict the future symbol.

More recently, recurrent neural networks and reservoir computers have been trained to recreate the output of any dynamical system. This is implemented via simple linear or logistic regression for reservoir computers [14] or via back-propagation through time for recurrent neural networks [15].

Often continuous-time data can be profitably represented as discrete-time data with a high sampling resolution. As such, one can essentially sample continuous-time, discrete-event data at high frequency and use any of the previously mentioned methods for predicting discrete-time data. Alternatively and more directly, one can represent continuous-time, discrete-event data as a list of continuous-valued *dwell times* and discrete symbols.

When it comes to continuous-time, discrete-event predictors, much effort has concentrated on continuous-time Markov processes with large state spaces [16,17,18]. In this, system states are wholly visible, but there are relatively sparse observations. As a result, we can impose structure on the kinetic rates (or intensity matrix) to simplify inference. Others considered temporal point processes, equivalent to the processes considered here. From them, the interevent interval distribution’s dependence on the history can be modeled parametrically [19] or using a recurrent neural network [20,21,22,23,24]. Though these are generative models, in theory they can be converted into predictive models [10,25] in which the dwell times are not generated, but instead kept track of for prediction. Furthermore, yet others used sequential Monte Carlo to make predictions from sampling distributions determined by these generative models [22,23].

We take a new approach: Infer continuous-time hidden Markov models with a particular (and advantageous) type of structure [10]. The models are designed to be a stochastic process’ “optimal predictor” [5,26] in that the model’s hidden state can be inferred almost surely from past data and in that the model’s hidden states are sufficient statistics—they provide the analyst with all the information needed to best predict the future and, in fact, to calculate all other desired process properties. This leads to the principal advantages of our method, as there is no need to sum over or infer a large number of latent variables; and even so, we comfortably learn models for and predict infinite-order Markov processes.

## 3. Background

We are given a sequence of symbols $x_{i}$ and durations $\tau_{i}$ of those events: a time series of the form $\ldots ,\left(x_{i},\tau_{i}\right),\left(x_{i+1},\tau_{i+1}\right),\ldots ,\left(x_{0},\tau_{0}^{+}\right)$. This list constitutes the data D. For example, animal behavioral data are of this kind: a list of activities and durations. The last seen symbol $x_{0}$ has been seen for a duration $\tau_{0}^{+}$. Had we observed the system for a longer amount of time, $\tau_{0}^{+}$ may increase. The possible symbols belong to a finite set $x_{i}\in A$, while the interevent intervals $\tau_{i}\in \left(0,\infty \right)$. We assume stationarity—the statistics of ${\left\{\left(x_{i},\tau_{i}\right)\right\}}_{i\in I}$ are invariant to the *start time*, where I is an interval of contiguous times.

Having specified the time series of interest, we turn to briefly introduce their representations—unifilar hidden semi-Markov models. Denoted M, we consider them as *generating* such time series [10]. The minimal such model consistent with the observations is the *ϵ-machine*. Underlying a unifilar hidden semi-Markov model is a finite-state machine with states *g*, each equipped with a dwell-time distribution $\phi_{g}\left(\tau \right)$, an emission probability p(x|g), and a function $\epsilon^{+}\left(g,x\right)$ that specifies the next hidden state when given the current hidden state *g* and the current emission symbol *x*.

**Figure 1:**
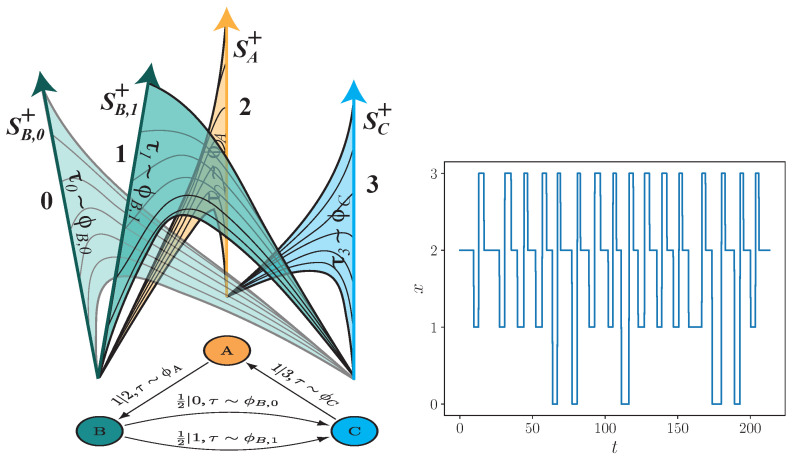
Unifilar hidden semi-Markov model (uhsMm): At left, two presentations of an example. (**Left bottom**) Generative three-state {A,B,C} model for a discrete-alphabet {0,1,2,3}, continuous-time stochastic process. Dwell times τ are drawn when transitioning between states, and the corresponding symbol is emitted for that amount of time, so that p|x,τ∼ϕ translates to symbol *x* is produced for a dwell time τ drawn from distribution ϕ with probability *p*. (**Left top**) Corresponding “conveyor belt” representation of the process generated by the model beneath. Conveyor belts represent the time since last symbol based on the height traveled along the conveyor belt; each conveyor belt has an event symbol. (**Right**) Example time series realization generated from the uhsMm, where $\phi_{A}$, $\phi_{B}$, and $\phi_{C}$ are inverse Gaussian distributions with (μ,λ) pairs of (1,2), (2,3), and (1,3), respectively.

This model generates a time series as follows: a hidden state *g* is randomly chosen; a dwell time τ is chosen according to the dwell-time distribution $\phi_{g}\left(\tau \right)$; an emission symbol *x* is chosen according to the conditional probability p(x|g); and we then emit the chosen *x* for duration τ. A new hidden state is determined via $\epsilon^{+}\left(g,x\right)$, and we further restrict possible next emissions to be different than the previous emission—a property that makes this model *unifilar*—and the procedure repeats. See Figure 1 for illustrations of a unifilar hidden semi-Markov model that is an ϵ-machine with three hidden states {A,B,C} which emits four events {0,1,2,3} with probabilistically varying durations.

## 4. Optimality

In this section, we restate the results of Ref. [10] in the language of Refs. [5,26] so that it is clear what kinds of models we would like to infer and why. Throughout this section, we assume stationarity.

We introduce a theorem that elucidates the representational power of the unifilar hidden semi-Markov models (ϵ-machines) discussed here that closely follows the proofs in Refs. [5,26]. Indeed, the only essential difference is that we are explicitly working with mixed random variables, whereas the initial references did not specify the type of random variable. Let $\overset{\leftarrow }{Y}$ represent the random variable for semi-infinite pasts and $\overset{\leftarrow }{y}$ its realization, and let $\vec{Y}$ represent the random variable for semi-infinite futures and $\vec{y}$ its realization. As described in Section 3, $\overset{\leftarrow }{y}$ is a list of past dwell times and past emitted symbols, ending with the present symbol and the time since last symbol. Furthermore, $\vec{y}$ is a list of future dwell times and future emitted symbols, starting with the present symbol and time to next symbol.

First, we define *causal states* as follows. Consider an equivalence relation on pasts: two pasts are considered equivalent, $\overset{\leftarrow }{y}{∼}_{\epsilon }{\overset{\leftarrow }{y}}^{\prime }$, if the conditional probability distributions over futures given the past are equivalent: $P\left(\vec{Y}|\overset{\leftarrow }{Y}=\overset{\leftarrow }{y}\right)=P\left(\vec{Y}|\overset{\leftarrow }{Y}={\overset{\leftarrow }{y}}^{\prime }\right)$. This equivalence relation partitions the set of pasts into causal states with associated random variable S and realization σ, such that $\sigma =\epsilon (\overset{\leftarrow }{y})$ is the causal state σ containing the past $\overset{\leftarrow }{y}$.

**Theorem 1**. *The causal states of a process generated by a hidden semi-Markov model are minimal sufficient statistics of prediction.*

**Proof.**  As the process is generated by a hidden semi-Markov model, we can meaningfully discuss the conditional probability distribution of futures given pasts. From the definition of the equivalence relation, we have that $P\left(\vec{Y}|\overset{\leftarrow }{Y}=\overset{\leftarrow }{y}\right)=P\left(\vec{Y}|S=\epsilon \left(\overset{\leftarrow }{y}\right)\right)$. Let ${\vec{Y}}^{T}$ denote futures of total duration *T*. It follows from $P\left(\vec{Y}|\overset{\leftarrow }{Y}=\overset{\leftarrow }{y}\right)=P\left(\vec{Y}|S=\epsilon \left(\overset{\leftarrow }{y}\right)\right)$ that $H\left[{\vec{Y}}^{T}|\overset{\leftarrow }{Y}\right]=H\left[{\vec{Y}}^{T}|S\right]$ for all *T*, from which it follows that $I\left[{\vec{Y}}^{T};\overset{\leftarrow }{Y}\right]=I\left[{\vec{Y}}^{T};S\right]$. (H[·], H[·|·], and I[·;·] are, respectively, the entropy, conditional entropy, and mutual information [27]). Hence, causal states S are sufficient statistics of prediction.

We then turn to the minimality of causal states. Since S is a sufficient statistic of prediction, the Markov chain $\vec{Y}\to S\to \overset{\leftarrow }{Y}$ holds. Consider any other sufficient statistic R of prediction. We are guaranteed the Markov chain $\vec{Y}\to S\to R$. Consider P(S=σ|R=r) and futures of length *T*. Note that:

$$\begin{array}{c} P\left({\vec{Y}}^{T}|R\;=\;r\right)\;=\;\;\sum_{\sigma }\;P\left(S\;=\;\sigma |R\;=\;r\right)P\left({\vec{Y}}^{T}|S\;=\;\sigma \right)\;. \end{array}$$

From the convexity of conditional entropy, we have that:

$$\begin{array}{c} H\left[{\vec{Y}}^{T}|R\;=\;r\right]\;\geq \;\sum_{\sigma }P\left(S\;=\;\sigma |R\;=\;r\right)H\left[{\vec{Y}}^{T}|S\;=\;\sigma \right]\;, \end{array}$$

with equality if P(S=σ|R=r) has support on one causal state σ. From the above inequality, we find that:

$$\begin{array}{cc} \sum_{r}P\left(R\;=\;r\right)H\left[{\vec{Y}}^{T}\right| & R\;=\;r]\;\geq \;\sum_{r,\sigma }\;P\left(R\;=\;r,S\;=\;\sigma \right)H\left[{\vec{Y}}^{T}|S\;=\;\sigma \right]\;. \end{array}$$

And so:

$$\begin{array}{cc} H[{\vec{Y}}^{T}|R]\; & \geq H[{\vec{Y}}^{T}|S] \end{array}$$

and:

$$\begin{array}{c} I\left[{\vec{Y}}^{T};R\right]\leq I\left[{\vec{Y}}^{T};S\right]\;, \end{array}$$

for any length *T*. This implies $I\left[\vec{Y};R\right]\leq I\left[\vec{Y};S\right]$. If R is a sufficient statistic, then equality holds; hence, from earlier comments, P(S|R=r) has support on only one causal state, and hence, H[S|R]=0.

A subtlety here is that S is a mixed discrete-continuous random variable and so, for the moment, we consider infinitesimal partitions of the aspect of S that tracks the time since last event and, then, take the limit as the partition size tends to 0, as is often done in calculations of entropy rate; see, e.g., Ref. [28]. From considering H[S,R], we find:

$$\begin{array}{cc} H[S]+H[R|S] & =H[R]+H[S|R]\\ H[S]+H[R|S] & =H[R]\\ H[S] & \leq H[R]\;, \end{array}$$

where we used the fact that H[R|S]≥0. We therefore established that if R is a minimal sufficient statistic of prediction, it must be equivalent to the causal states S. □

In what follows, we relate causal states to the hidden states of *minimal unifilar* hidden semi-Markov models, relying heavily on Ref. [26]. Indeed, the only difference between the proofs is that here we have explicitly identified g,x,τ as the causal states.

**Theorem 2**. *The hidden states of the minimal unifilar hidden semi-Markov model—i.e., typically g, x, and τ—are causal states.*

**Proof.**  Since a detailed proof is given in Ref. [26], we state the issues somewhat informally. A minimal unifilar hidden semi-Markov model has two key properties:

- *Unifilarity*: if the current hidden state and next emission are known, then the next hidden state is determined; and
- *Minimality*: minimal number of states (or generative complexity [29]) out of all unifilar generators consistent with the observed process.

Let G be the random variable denoting the hidden state. Clearly $\overset{\leftarrow }{Y}\to G\to \vec{Y}$ for any hidden Markov model. The unifilarity of the model guarantees that we can almost surely determine the hidden state of the model given the past (i.e., that the hidden state is a function of the past) and, hence, $G\to \overset{\leftarrow }{Y}\to \vec{Y}$. The Data Processing Inequality applied twice implies that $I\left[\vec{Y};\overset{\leftarrow }{Y}\right]=I\left[\vec{Y};G\right]$, and so the hidden state is a sufficient statistic of prediction. As we are focusing on the *minimal* unifilar model, G is the minimal sufficient statistic of prediction, and so there is an isomorphism between the machine constructed from S and the minimal unifilar machine. Since the minimal unifilar machine typically has hidden states g,x,τ (with exceptions seen when the dwell time distributions have special structure [10]), these are causal states. □

Theorem 2 provides the inspiration for the algorithms that follow.

## 5. Continuous Time-Bayesian Structural Inference (CT-BSI) and Comparison Algorithms

We investigate and then provide algorithms for three tasks: model inference, calculating the differential entropy rate, and predicting future symbols. Our main claim is that restricting attention to a special type of discrete-event, continuous-time model—the unifilar hidden semi-Markov models or ϵ-machine—renders all three tasks markedly easier since the model’s hidden states are minimal sufficient statistics of prediction, based on Theorem 2. The restriction is, in fact, not much of one, as the ϵ-machines can finitely represent an unboundedly larger set of processes compared to those generated by Markov and semi-Markov models.

### 5.1. Inferring Optimal Models of Unifilar Hidden Semi-Markov Processes

The unifilar hidden semi-Markov models described earlier can be parameterized. Let M refer to a model—in this case, the underlying topology of the finite-state machine and neural networks defining the density of dwell times. Let θ refer to the model’s parameters; i.e., the emission probabilities and the parameters of the neural networks. Furthermore, let D refer to the data; i.e., the list of emitted symbols and dwell times. Ideally, to choose a model we maximize the posterior distribution by calculating $\arg\max_{M}\mathrm{Pr}\left(M|D\right)$ and select parameters of that model via maximum likelihood: $\arg\max_{\theta }\mathrm{Pr}\left(D|\theta ,M\right)$.

In the case of discrete-time unifilar hidden Markov models, Strelioff and Crutchfield [8] described the Bayesian framework for inferring the best-fit model and parameters. More than that, Ref. [8] calculated the posterior analytically, using the unifilarity property to ease the mathematical and statistical burdens. Analytic calculations in continuous-time may be possible, but we leave that for a future endeavor. We instead turn to a variety of approximations, still aided by the unifilarity of the inferred models. Again, the unifilarity property allows for tractable inference of models that are typically infinite-order Markov.

The main such approximation is our use of the Bayesian information criterion (BIC) [9], which allows us to do approximate model selection despite not being to exactly calculate the posterior. Maximum a posteriori model selection is performed via:

(1) $$\begin{array}{cc} \mathrm{BIC} & =\frac{k_{M}}{2}\log\left|D\right|-\max_{\theta }\log\mathrm{Pr}\left(D|\theta ,M\right)\\ M^{*} & =\arg\min_{M}\mathrm{BIC}\;, \end{array}$$

where $k_{M}$ is the number of parameters θ. (Note that this is a factor of two off of the usual formulation, with no changes to the inferred model or parameters.) As we are planning to select between models, $k_{M}$ will depend on the model. More complex models have more parameters, owing to a greater number of states. To choose a model, then, we must calculate not only the parameters θ that maximize the log likelihood, but the log likelihood itself.

We make one further approximation for tractability involving the uhsMm start state $s_{0}$, for which:

$$\begin{array}{c} \mathrm{Pr}\left(D|\theta ,M\right)=\sum_{s_{0}}\pi \left(s_{0}|\theta ,M\right)\mathrm{Pr}\left(D|s_{0},\theta ,M\right)\;. \end{array}$$

Since the logarithm of a sum has no simple expression, and since with more data our estimation of the probability distribution over start states should be highly peaked, we approximate:

$$\begin{array}{c} \max_{\theta }\log\mathrm{Pr}\left(D|\theta ,M\right)\approx \max_{s_{0}}\max_{\theta }\log\mathrm{Pr}\left(D|s_{0},\theta ,M\right)\;. \end{array}$$

If it is possible to infer the start state from the data—which is the case for all the models considered here—then the likelihood should overwhelm the prior’s influence. Our strategy, then, is to choose parameters θ that maximize $\max_{s_{0}}\log\mathrm{Pr}\left(D|s_{0},\theta ,M\right)$ and to choose the model M that minimizes the BIC in Equation (1). This constitutes inferring a model that explains the observed data as well as possible.

What remains to be done, therefore, is approximating $\max_{s_{0}}\max_{\theta }\log\mathrm{Pr}\left(D|s_{0},\theta ,M\right)$. The parameters θ of any given model include $p(s^{\prime },x|s)$, the probability of emitting *x* when in state *s* and transitioning to state $s^{\prime }$, and $\phi_{s}\left(t\right)$, the interevent interval distribution of state *s*. Using the unifilarity of the underlying model, the sequence of *x*’s when combined with the start state $s_{0}$ translate into a *single* possible sequence of hidden states $s_{i}$. We have:

(2) $$\mathrm{Pr}\left(D|s_{0},\theta ,M\right)=\prod_{i}p\left(s_{i},x_{i}|s_{i-1}\right)\phi_{s_{i}}\left(\tau^{\left(s_{i}\right)}\right).$$

As such, one can show that:

(3) $$\begin{array}{c} \log\mathrm{Pr}\left(D|s_{0},\theta ,M\right)=\sum_{s}\sum_{j}\log\phi_{s}\left(\tau_{j}^{\left(s\right)}\right)+\sum_{s,x,s^{\prime }}n\left(s^{\prime },x|s\right)\log p\left(s^{\prime },x|s\right)\;, \end{array}$$

where $n(s^{\prime },x|s)$ is the number of times we observe an emission *x* from a state *s* leading to state $s^{\prime }$ and where $\tau_{j}^{\left(s\right)}$ is any interevent interval produced when in state *s*. It is relatively easy to analytically maximize with respect to $p(s^{\prime },x|s)$, including the constraint that $\sum_{s^{\prime },x}p\left(s^{\prime },x|s\right)=1$ for any *s*. We find that:

(4) $$\begin{array}{c} p^{*}\left(s^{\prime },x|s\right)=\frac{n(s^{\prime },x|s)}{n(s)}\;, \end{array}$$

where n(s) is the number of times the model visits state *s*.

Now, we turn to approximate the dwell-time distributions $\phi_{s}\left(t\right)$. In theory, a dwell-time distribution can be any normalized nonnegative function. With sufficient nodes artificial neural networks can represent any continuous function. We therefore represent $\phi_{s}\left(t\right)$ by a relatively shallow (here, five-layer) artificial neural network in which nonnegativity and normalization are enforced as follows:

- The second-to-last layer’s activation functions are ReLus (max(0,x) and so have nonnegative output) and the weights to the last layer are constrained to be nonnegative; and
- The output is the last layer’s output divided by a numerical integration of the last layer’s output.

A wider (25 nodes instead of 15) and shallower (one less layer) network is measurably worse, not shown here.

**Figure 2:**
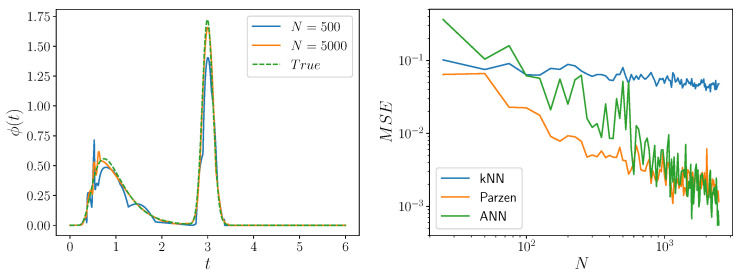
**Estimated dwell-time density function for varying numbers of samples.** (**Left**) Inferred density function using the neural network described here compared to the true density function (dotted, green) when given 500 samples (blue) and 5000 samples (orange). As the sample size increases, the inferred density function better approximates ground truth. An interevent interval distribution with two modes was arbitrarily chosen by setting ϕ(τ) to a mixture of two inverse Gaussians. (**Right**) Mean-squared error between the estimated density and the true density as we use more training data for three different estimation techniques. The green line denotes the ANN algorithm introduced here, in which we learn densities from a neural network, running with five different seeds and choosing the one with the lowest MSE; the blue line denotes the *k*-nearest neighbors algorithm [9,31]; and the orange line gives Parzen-window estimates [9,32]. Our new method is competitive with these two standard methods for density estimation and quantitatively equivalent to the Parzen estimator at moderate to large samples.

The log likelihood $\sum_{j}\log\phi_{s}\left(\tau_{j}^{\left(s\right)}\right)$ determines the cost function for the neural network, so that the output of the neural network for an array of input dwell times is an equally-sized array of estimated densities. Then, the neural network can be trained using typical stochastic optimization methods. (Here, we use Adam [30].) The neural network output can successfully estimate the interevent interval density function, given sufficient samples, within the interval for which there is data. See Figure 2. Note, though, that if samples are too sparsely spaced, estimation of ϕ(τ) does not happen correctly. Outside this interval, however, the estimated density function is not guaranteed to vanish as t→∞, and it can even grow. Stated differently, the neural networks considered here are good interpolators, but can be bad extrapolators. As such, the density function estimated by the network is taken to be 0 outside the interval over which there is data.

To the best of our knowledge, this is a new approach to density estimation, referred to as *ANN* here. A previous approach to density estimation using neural networks learned the cumulative distribution function [33]. Another more popular approach expresses the interevent interval as $\lambda \left(t\right)e^{-\int^{t}\lambda \left(s\right)ds}$, where λ(t) is the intensity function. Analysts then either parameterize the intensity function or use a recurrent neural network [20,21,22,23] to model λ(t). Note that the log-likelihood for this latter approach also involves numerical integration, but this time, of the intensity function. This integral accounts for the probability of nonevents. Some assume a particular form for the interevent interval and fit parameters of the functional form to data [19], while others treat estimation of the cumulative density function or the dwell time distribution as a regression problem [34,35]. More traditional approaches to density estimation include *k*-nearest neighbor estimation techniques and Parzen-window estimates, both of which need careful tuning of hyperparameters (*k* or *h*) [9]. They are referred to here as *kNN* and *Parzen*, respectively.

We compare ANN, kNN, and Parzen approaches to inferring an interevent interval density function that we have chosen, arbitrarily, to be the mixture of inverse Gaussians shown in Figure 2 (Left). The *k* in *k*-nearest neighbor estimation is chosen according to Ref. [31]’s criterion and *h* is chosen to maximize the pseudo-likelihood [32]. Note that, as Figure 2 (Right) shows, ANN is not a superior approach to density estimation in terms of minimization of mean-squared error, but it is parametric, so that BIC model selection can be used.

The approach taken here is certainly not the only promising approach one can invent. Future work will investigate both the efficacy of parametrizing the intensity function rather than the interevent interval density function [20,21,22,23] and the benefits of learning normalizing flows [36].

**Figure 3:**
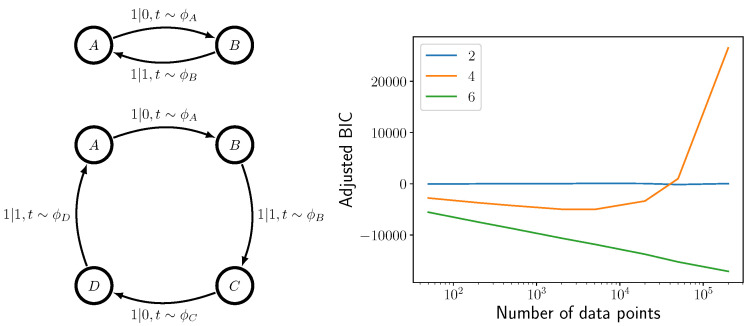
Model order selection. (**Left**) Two-state model (**top**) and four-state uhsMm (**bottom**) for binary-alphabet, continuous-time data. (**Right**) Adjusted BIC, or $-BIC+\left(1.4*N+698*\log N-5.5\right)$, as a function of sample size for the two-state, four-state, and six-state uhsMms at left. (The six-state uhsMm is not shown.) Adjusted BIC is shown only to make it clearer where the four-state machine is deemed more probable than the two-state machine. Smaller BIC (higher Adjusted BIC) implies a higher posterior probability and so a better fit.

To test our new method for density estimation—that is, training a properly normalized ANN—we generated a trajectory from the unifilar hidden semi-Markov model shown in Figure 3 (Left) and used BIC to select the correct model. As BIC is a penalty for a larger number of parameters minus a log likelihood, a smaller BIC suggests a higher posterior probability. With very little data, the two-state model shown in Figure 3 is deemed to be the most likely generator. However, as sample size increases, the correct four-state model eventually takes precedence. See Figure 3 (Right). The six-state model was never deemed more likely than a two-state or four-state model.

Note that although this methodology might be extended to nonunifilar hidden semi-Markov models with addition of Monte Carlo sampling or inference of the most likely trajectory of hidden states, unifilarity allowed for easily computable and unique identification of dwell times with states in Equation (3). Though Ref. [20] led to a larger log likelihood (∼−6000 versus ∼−900) for the same amount of data (N=2000) for the four-state model, the interpretability of the unifilar hidden semi-Markov model may be higher.

### 5.2. Improved Differential Entropy Rates

One benefit of unifilar hidden semi-Markov models is that they directly lead to explicit formulae for information generation—the differential entropy rate [10]—for a wide class of infinite causal-state processes like those generated by uhsMms. Generally, entropy rates measure a process’ inherent randomness [37] and so they are a fundamental characteristic. As such, much effort has been invested to develop improved entropy-rate estimators for complex processes [38,39,40,41] since they aid in classifying processes [42]. We now ask how well one can estimate the entropy rate from finite data for continuous-time, discrete-event processes. In one sense, this is a subtle problem: estimating a property of an effectively infinite-state process from finite data. Although, from another perspective, we should be able to estimate a scalar property of an infinite-dimensional object from finite data with high accuracy [40].

Compounding this, infinite-state processes or not, differential entropy rates are difficult to calculate directly from data, since the usual method calculates the entropy of trajectories of some length *T*, dividing by *T* to get a rate:

$$\begin{array}{c} h_{\mu }=\lim_{T\to \infty }T^{-1}H\left[{\vec{\left(x,\tau \right)}}_{0:T}\right]\;. \end{array}$$

A better estimator, though, is the following [37]:

$$\begin{array}{c} h_{\mu }=\lim_{T\to \infty }\frac{d}{dT}H\left[{\vec{\left(x,\tau \right)}}_{0:T}\right]\;, \end{array}$$

which is the slope of the graph of $H[{\vec{\left(x,\tau \right)}}_{0:T}]$ versus *T*.

As the entropy of a mixed random variable of unknown dimension, this entropy appears difficult to estimate from finite data. To calculate $H[{\vec{\left(x,\tau \right)}}_{0:T}]$, we use an insight from Ref. [43] and condition on the number of events *N*:

$$\begin{array}{c} H\left[{\vec{\left(x,\tau \right)}}_{0:T}\right]=H\left[N\right]+H\left[{\vec{\left(x,\tau \right)}}_{0:T}|N\right]\;. \end{array}$$

We then break the entropy into its discrete and continuous components:

$$\begin{array}{c} H\left[{\vec{\left(x,\tau \right)}}^{T}|N=n\right]=H\left[x_{0:n}|N=n\right]+H\left[\tau_{0:n}|x_{0:n},N=n\right] \end{array}$$

and use the *k*-nearest-neighbor entropy estimator [44] to estimate $H[\tau_{0:n}|x_{0:n},N=n]$, arbitrarily choosing k=3. (Other *k*s did not substantially affect results.) We estimate both $H[x_{0:n}|N=n]$ and H[N] using plug-in entropy estimators, as the state space is relatively well-sampled. We call this estimator *model-free*, in that we need not infer a state-based model to calculate the estimate.

We introduce a model-based estimator, for which we infer a model and then use the inferred model’s differential entropy rate as the differential entropy rate estimate. To calculate the differential entropy rate from the inferred model, we use a plug-in estimator based on the formula in Ref. [10]:

(5) $$\begin{array}{c} \hat{h_{\mu }}=-\sum_{s}\hat{p}\left(s\right)\int_{0}^{\infty }{\hat{\mu }}_{s}{\hat{\phi }}_{s}\left(t\right)\log{\hat{\phi }}_{s}\left(t\right)dt\;, \end{array}$$

where the sum is over the model’s internal states. For certain special processes with long tails on the dwell time distribution, the estimated entropy rate might not exist, but this will never be a practical problem if typical kernel density estimates are used. The parameter $\mu_{s}$ is simply the mean interevent interval out of state *s*: $\mu_{s}=\int_{0}^{\infty }t{\hat{\phi }}_{s}\left(t\right)dt$. We find the distribution $\hat{p}\left(s\right)$ over internal states *s* by solving the linear Equations [10]:

(6) $$\begin{array}{c} p\left(s\right)=\sum_{s^{\prime }}\frac{\mu_{s^{\prime }}}{\mu_{s}}\frac{n_{s^{\prime }\to s}}{n_{s^{\prime }}}p\left(s^{\prime }\right)\;. \end{array}$$

We use the MAP estimate of the model as described previously and estimate the interevent interval density functions $\phi_{s}\left(t\right)$ using a Parzen-window estimate, better known as a kernel density estimate, given that those proved to have lower mean-squared error than the neural network density estimation technique in the previous subsection. The smoothing parameter *h* was chosen to maximize the pseudo-likelihoods [32]. In other words, we have to use neural network density estimation to choose the model so that we have a parametric method that works with BIC. However, with the model in hand, we use Parzen-window estimates to estimate the density for purposes of estimating entropy rate. A full mathematical analysis of the bias and variance is beyond the present scope.

**Figure 4:**
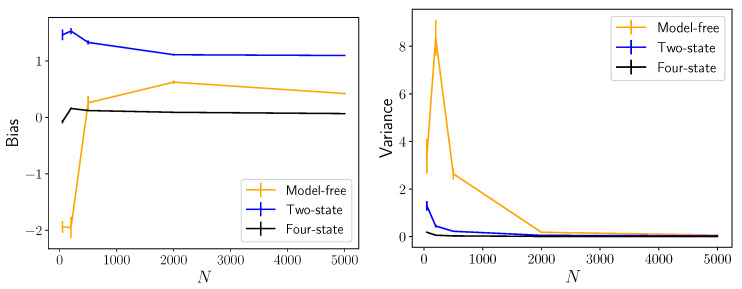
Model-free versus model-based entropy rate estimators. Synthetic dataset generated from Figure 3 (**top**) with $\phi_{A}\left(t\right)=\phi_{D}\left(t\right)$ as inverse Gaussians with mean 1 and scale 5 and with $\phi_{B}\left(t\right)=\phi_{C}\left(t\right)$ as inverse Gaussians with mean 3 and scale 2. The ground truth entropy rate from the formula in [10] is 1.85 nats. In orange, the model-free estimator (combination of plug-in entropy estimator and kNN [44] entropy estimators) described in the text. In blue, the model-based estimator assuming a two-state model, i.e., the top left of Figure 3. In black, the model-based estimator assuming a four-state model, i.e., the bottom left of Figure 3. Lines denote the mean bias (**left**) or standard deviation (**right**) in entropy rate estimates, and error bars show estimated standard deviation in such. The model-free method has much higher bias and variance than both model-based methods.

Figure 4 compares the model-free method (*k*-nearest neighbor entropy estimator) and the model-based method (estimation using the inferred model and Equation (5)) as a function of the length of trajectories simulated for the model. In Figure 4, the blue data points describe what happens when the most likely (two-state) model is used for the model-based plug-in estimator of Equation (5), whereas the black data points describe what happens when the correct four-state model is used for the plug-in estimator. That is, for the two-state model the estimate given by Equation (5) is based on the *wrong model* and, hence, leads to a systematic overestimate of the entropy rate (nonzero bias) with unreasonable confidence (low variance). When the correct four-state model is used for the plug-in estimator in Figure 4, the model-based estimator has much lower bias *and* variance than the model-free method.

To efficiently estimate the past-future mutual information or *excess entropy* [37,45,46], an important companion informational measure, requires models of the time-reversed process. A sequel will elucidate the needed retrodictive representations of unifilar hidden semi-Markov models, which can be determined from the “forward” unifilar hidden semi-Markov models. This and the above methods lead to a workable excess entropy estimator.

### 5.3. Improved Prediction with Causal States

A wide array of techniques have been developed for discrete-time prediction, as described in the introduction. Using dwell times and symbols as inputs to a recurrent neural network, for example, we can develop continuous-time techniques that build on these discrete-time techniques. However, we will demonstrate that we gain a surprising amount by first identifying continuous-time causal states.

The first prediction method we call *predictive ANN* (PANN) (risking confusion with the ANN method for density estimation described earlier) takes as input $\left(x_{-n+1},\tau_{-n+1}\right),\ldots ,\left(x_{0},\tau_{0}^{+}\right)$ into a feedforward neural network that is relatively shallow (six layers) and somewhat thin (25 nodes). (Other network architectures were tried with little improvement.) The network weights are trained to predict the emitted value *x* at time *T* later based on a mean-squared error loss function. For this to work, the neural network must predict the hidden state *g* from the observed data. This can be accomplished if the dwell-time distributions of the various states are dissimilar. Increases in *n* can increase the network’s ability to correctly predict its hidden state and thus predict future symbols. This assumes sufficient data to avoid overfitting; here, *n* is chosen via cross-validation.

The second method, called *RNN*, takes $\left(x_{-n+1},\tau_{-n+1}\right),\ldots ,\left(x_{0},\tau_{0}^{+}\right)$ as input to a *long short-term memory* (LSTM) neural network [47,48]. (Though any recurrent neural network could have been chosen). *n* was chosen by cross-validation. The LSTM is tasked to produce an estimate of *x* at time *T* subject to a mean-squared error loss function, similar to the PANN method.

For both PANN and RNN, a learning rate was chosen an order of magnitude smaller than the learning rate that led to instability. In fact, a large number of learning rates that were orders of magnitude smaller than the critical learning rate were tried.

The third method is our *Continuous-Time Bayesian Structural Inference* algorithm, labeled CT-BSI. It preprocesses input data using an inferred unifilar hidden semi-Markov model so that each time step is associated with a hidden state *g*, a time since last symbol change $\tau_{0}^{+}$, and a current emitted symbol $x_{0}$. In discrete-time applications, there is an explicit formula for the optimal predictor in terms of the ϵ-machine’s labeled transition matrix. However, for continuous-time applications, there is no closed-form expression, and so we use a *k*-nearest neighbor estimate of the data a time *T* into the future. More precisely, we find the *k* closest data points in the training data to the data point at present in the hidden state space with the condition that *g* and *x* are identical, and estimate $x_{T}$ as the average of the future data points in the training set. In the limit of infinite data in which the correct model is identified, for correctly-chosen *k*, this method outputs an optimal predictor. We choose *k* via cross-validation.

All of these methods were developed as though we were attempting regression, but could easily be used for classification tasks. PANN and RNN could have a softmax output layer for choosing the next symbol, while CT-BSI could choose the next symbol seen by majority vote of the future of *k* nearest neighbors in the hidden state space. Note that while the output might be unordered, the hidden state space can be ordered due to τ being a real number.

The synthetic dataset is generated from Figure 3 (Left, bottom) with $\phi_{A}\left(t\right)=\phi_{D}\left(t\right)$ as inverse Gaussians with mean 1 and scale 5 and with $\phi_{B}\left(t\right)=\phi_{C}\left(t\right)$ as inverse Gaussians with mean 3 and scale 2. We chose these means and scales so that it would be easier, in principle, for the non-uhsMm methods (i.e., PANN and RNN) to implicitly infer the hidden state (*A*, *B*, *C*, and *D*). Given the difference in dwell time distributions for each of the hidden states, such implicit inference is necessary for accurate predictions.

**Figure 5:**
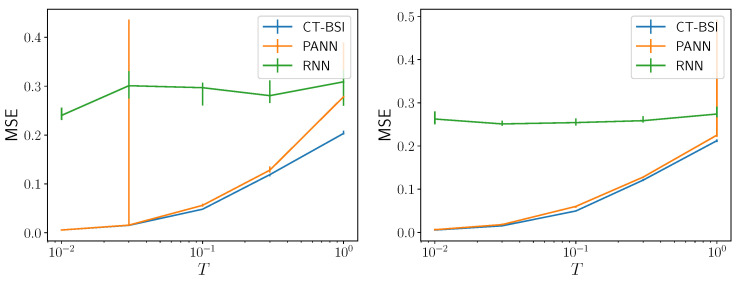
Prediction. Mean-squared prediction error for the data point a time *T* away based on training with 500 (**Left**) and 5000 (**Right**) data points. 3000 epochs were used to train the ANN. 68% confidence intervals are shown. The data generating uhsMm is that in Figure 3 (**Left**, **bottom**). The CT-BSI method infers the internal state of the unifilar hidden semi-Markov model; the PANN method uses the last *n* data points $\left(x_{i},\tau_{i}\right)$ as input into a feedforward neural network; and the RNN method uses the past $\left(x_{i},\tau_{i}\right)$ as input to an LSTM.

Figure 5 demonstrates that CT-BSI outperforms the feedforward neural network (PANN) and the recurrent neural network (RNN). The corresponding mean-squared errors for the three methods are shown there for two different dataset sizes. Note that mean-squared error might not be calculable for processes whose observables are non-binary. Different network architectures, learning rates, and number of epochs were tried; the results shown are typical. We employed a *k*-nearest neighbor estimate on the causal states (i.e., the uhsMm’s internal state) to predict the future symbol. Overall, CT-BSI requires little hyperparameter tuning and outperforms substantially more compute-intensive feedforward (PANN) and recurrent neural network (RNN) algorithms.

The key here is trainability: It is difficult to train RNNs to predict these sequences, even though RNNs are intrinsically more expressive than PANNs. As such, they perform measurably worse. PANNs work quite well, but as shown in Figure 5 (Left), with small amounts of data, PANNs can sporadically learn wildly incorrect mappings to future data. This occurs at intermediate timescales: See the the marked increase in the size of the confidence interval at $T=2\times 10^{-2}$ in Figure 5 (Left). However, this also occurs at long timescales with larger data sets: See the large increase in mean MSE from the superior performance of CT-BSI at $T=10^{0}$ in Figure 5 (Right). CT-BSI, in contrast, learns low variance predictions with lower MSE than both RNNs and PANNs. The mean-squared errors for CT-BSI are comparable to an approximation of the optimal mean-squared error based on a discrete-time approximation of the continuous-time unifilar hidden semi-Markov model.

## 6. Discussion

We introduced the Continuous-Time Bayesian Structural Inference (CT-BSI) algorithm to infer the causal states [5] of continuous-time, discrete-event processes, showing that it outperforms suitably generalized neural network architectures. This leveraged prior groundwork on discrete-time, discrete-event processes [10] and Bayesian Structural Inference for processes generated by finite-state HMMs [8]. This led to a natural new entropy-rate estimator that uses a process’ causal states and a new predictor based on causal states that is more accurate than competitors. Finally, and key to applications, compared to the neural network competitors CT-BSI’s inferred causal states and ϵ-machine give an explicit and interpretable mechanism for a process’ generator. Note that these tools can apply even when there is no ordering on the observed symbols.

The major challenge with applying these tools is model mismatch—the true or a closely-related model might not be inferred. This can lead to inaccurate estimations of the entropy rate and also to inaccurate predictions. However, as discussed, if sufficient data is available, a more complex model will be favored, which might be closer to ground truth. Additionally, we conjecture that the processes generated by unifilar hidden semi-Markov models are dense in the space of all possible stationary continuous-time, discrete-event processes. If true, the restriction to unifilar models is not a severe limitation, as there will always be nearby unifilar model with which to estimate and predict. A second issue—which also plagues the discrete-time, discrete-event Bayesian structural inference algorithm [8]—is searching over all possible topologies of unifilar hidden semi-Markov models [49]. Circumventing both of these challenges suggests exploring nonparametric Bayesian approaches [13].

The new inference, estimation, and prediction algorithms can be used to analyze continuous-time, discrete-event processes—a broad class spanning from seismic time series to animal behavior—leading to reliable estimates of the intrinsic randomness of such complex infinite-memory processes. Future efforts will delve into improved estimators for other time series information measures [50], using model selection criteria more accurate than BIC to identify MAP models, and enumerating the topology of all possible uhsMm models for nonbinary alphabets [49]. In addition, future work will compare the predictive features of Ref. [20] to the predictive features inferred here.

## Data Availability

The data used will be made available upon reasonable request to the first author.
